# Molecular Characterization of the Full Muscovy Duck Parvovirus, Isolated in Guangxi, China

**DOI:** 10.1128/genomeA.01249-14

**Published:** 2014-12-11

**Authors:** Hong Zhao, Zhixun Xie, Liji Xie, Xianwen Deng, Zhiqin Xie, Sisi Luo, Li Huang, Jiaoling Huang, Tingting Zeng

**Affiliations:** a College of Animal Science and Technology, Guangxi University, Nanning, Guangxi, China; b Guangxi Key Laboratory of Animal Vaccines and Diagnostics, Guangxi Veterinary Research Institute, Nanning, Guangxi, China

## Abstract

We report the complete genomic sequence of the full Muscovy duck parvovirus (MDPV) strain, designated GX2011-5, isolated from a Muscovy duck in Guangxi Province, China. The complete genomic sequence was 5,132 bp in length and contained two major open reading frames encoding a 1,844-nucleotide (nt) nonstructural protein and a 2,199-nt capsid protein. Comparison of the complete sequence of GX2011-5 with other published sequences of Muscovy duck parvovirus revealed that this strain exhibited 90.4% to 95.1% sequence homology. This report will advance our understanding of the epidemiology and molecular characteristics of MDPV in the Muscovy duck population in Guangxi, China.

## GENOME ANNOUNCEMENT

Muscovy duck parvovirus (MDPV) is a single-stranded DNA virus that belongs to the family *Parvoviridae* (1). Muscovy ducklings under 3 weeks of age are the most susceptible to contracting MDPV infection. The clinical signs of the disease included watery diarrhea, wheezing, and locomotor dysfunction. Once these signs are observed, death occurs rapidly. Although the mortality is much lower in ducks infected after 3 weeks of age, the production performance of ducks that survive the infection is reduced because of abnormal feathering and growth retardation (2, 3, 4). To date, many MDPVs have been isolated and sequenced; however, there are few reports of strains isolated from Guangxi (5, 6, 7). The aim of this study is to obtain the full genomic sequence of an MDPV strain isolated from Muscovy ducks.

In May 2011, MDPV was isolated from a commercial Muscovy duck farm during an outbreak of an infectious disease. The clinical symptoms manifested with severe watery diarrhea and locomotor dysfunction in ducklings in the city of Yulin in Guangxi Province in southern China. Subsequently, nucleotide sequences of MDPV were amplified through PCR. The amplified products were purified and cloned into the pMD18-T vector (TaKaRa) and then sequenced (TaKaRa, Dalian, China) (8, 9, 10). The sequences were assembled and manually edited to produce the final genome sequence. The isolated virus was named duck/Guangxi/05/2011 (GX2011-5).

The full-length genome sequence of GX2011-5 was 5,132 nucleotides (nt) in length, containing two major open reading frames (ORFs). The left ORF (ORF1, 1,884 nt) encodes pleiotropic regulatory proteins (NS1, NS2) and 627 amino acids (aa), the right ORF (ORF2, 2,199 nt) encodes the viral capsid proteins (VP1, VP2, VP3) and 732 amino acids (aa), Analysis of the potential glycosylation sites of the surface proteins revealed four potential N-glycosylation sites in NS1 (150-153, 225-228, 360-363, and 433-436) and four potential N-glycosylation sites in VP1 (219-222, 331-334, 582-585, and 703-705) (11, 12).

Compared to the genome sequences of isolates of MDPV from different areas, the nucleotide sequence of GX2011-5 shared 95.1% homology with the sequence of the strain SAAS-SHNH (GenBank accession no. KC171936.1; origin, Shanghai, China), it shared 90.4% homology with the sequence of the strain FM (GenBank accession number NC_006147.2; origin, France). The NS1 and VP1 genes of GX2011-5 shared 97.9 to 99.5% and 88.7 to 99.3% homology, respectively, with the NS1 and VP1 sequences of the strains SAAS-SHNH, FM, P (GenBank accession no. JF26997.1; origin, Fujian, China), P1 (GenBank accession n. JF26998.1; origin, Fujian, China), and X75093 (GenBank accession no. X75093.1; origin, France). These results indicate that the NS1 gene is much more conserved than the VP1 gene (13).

In conclusion, the study of the whole-genome sequence of MDPV requires further investigation to assess the epidemiology and evolution of MDPV, and such studies may help to elucidate the mechanisms of virus replication and pathogenesis.

### Nucleotide sequence accession number.

The complete genome sequence of the Muscovy duck parvovirus isolate described here has been deposited in GenBank under the accession no. KM093740.
